# 

**Published:** 1889-12

**Authors:** 


### INDEX

TO THE

### HOMŒOPATHIC PHYSICIAN.

### VOLUME IX.

PAGE

Acetic Acid, . . . . . . . . . . . . 203, 207
Aconite, . . 10, 78, 142, 198, 201, 235, 246, 247, 337, 423, 436
Actea racemosa, . . . . . . . . . 209
Acute and Chronic Tonsillitis. E. J. Lee, M. D., . . . . . . . . . . . . . 77
Address of Dr. Geo. Wigg, Extract from the, . . . . . . . . . . . . . 259
Æsculus Hipp., . . . . . . . . . . . 207
Æthusa, . . . . . . . . . . 173, 340, 348, 424
Æthusa Cynapium, Note on. E. W. Berridge, M. D., . . . . . . . . . . 353
Agaricus, . . . . . . . . . . . 43, 208, 424
A. I H., The, . . . . . . . . . . . . . 48
Ailanthus, . . . . . . . . . . . . . . 183
Alcholisme et Criminalité, traitement Médical de l'ivrognerie et de l'ivresse. Par le Dr. Gallavardin. Review of, . . . . . . . . . . . . . 215
Allen, John V. Repertory to Labor and After Pains, . . . . . . . . . . 291
Allium Cepa, . . . . . . . . . . . . . 32
Allopathic Ignorance and Arrogance. B. Fincke, M. D., . . . . . . . . . . 212
Alumina in Infantile Paralysis, Action of. E. W. Berridge, M. D., . . . . . 243
Alumina, Provings of. E. W. Berridge, M. D., . . . . . . . . . . . . 351
Alumina, . . . . . . . . . . 242, 250, 419
American Institute, . . . . . . . . . 48
American Institute. Session of 1889, . 109
American Public Health Asso. Meeting, . . . . . . . . . . . . . . . 312
Ammonium carb., . . . . . . . . . . 79
Amygdala persica, . . . . . . . . . . 80
Amyl-nitrite, . . . . . . . . . . . . 424
Anacardium, . . . . . . . . . . . 246, 247
Annals of Surgery, The. Notice of, 168, 264
Antimonium tart., . . . . . . . . . 25, 180
Anti-Psoric Remedies, Extract from Dr. C. v. Bœnninghausen's preface to his repertory of the. Trans. by F. H. Lutze, M. D., . . . . . . . . 119
Apis, 80, 91, 173, 183, 235, 316, 325. 332, 398, 417, 419, 424
Appreciative Friends, . . . . . . . . 108
April Number, The, . . . . . . . . . 198
Argentum, . . . . . . . . . . . . . 253
Argentum-nit., . . . . . . . . . . 424, 434
Arnica, . 142, 198, 209, 298, 331, 354, 370, 424
Arnica or Calendula, . . . . . . . . . 369

PAGE

Arsenicum, 19, 179, 181, 184, 241, 247, 248, 251, 323, 324, 419, 425
Arsen. jod., . . . . . . . . . . . . . 184
Arum. triphyllum, . . 80, 184, 235, 246, 346
Atrophia sulph., . . . . . . . . . . . 207
Aurum, . . . . . . . . . . . . . . 20, 248

Badiaga, . . . . . . . . . . . . . . . 379
Baker, W. H., Sec. Proceedings of the Rochester Hahnemannian Society, . . . . . . . . . . . . . 25, 72
Ballard, E. A., M. D. Bureau of Materia Medica and Provings, I. H. A. 110
Ballard, E. A. A Verification, . . . . 259
Baptisia Tinctora. G. W. Sherbino, M. D. . . . . . . . . . . . . . . . 330
Baptisia, . . . . . . . 80, 179, 184, 209, 331
Baryta-carb., . . . . . . . . 19, 81, 91, 190
Baryta-mur., . . . . . . . . . . . . . 81
Baylies, B. L. B. Fracture of a rib, with immediate aggravation and speedy relief from Hypericum, . . . 198
Baylies, B. L. B., M. D. Medicinal aid in Parturition, . . . . . . . . . . . 142
Bell, James B. Dioscorea. Therapeutics of the Throat, . . . . . . . 40
Bell, James B. Indium, Therapeutics of the Throat, . . . . . . . . . . 40
Belladonna, 11, 81, 140, 143, 147, 180, 184, 199, 237, 238, 246, 247, 314, 325, 326, 336, 344, 425, 433
Benzoic-acid, . . . . . . . . . . . . 30
Berberis Vulgaris, Constipation of. John L. Ferson, M. D., . . . . . . . 174
Berberis, . . . . . . . . . . . . . . 355
Berridge, E. W., M. D. Note on Æthusa Cynapium, . . . . . . . . 353
Action of Alumina in Infantile Paralysis, . . . . . . . . . . . . 243
Provings of Alumina, . . . . . . 350
Case of Sunstroke, . . . . . . . . 451
A Cinnabar Case, . . . . . . . . . 399
Clinical Cases, . . . . . . . . 146, 192
A Clinical Conversation, . . . . . 347
Fragmentary Provings, . . . . . . 60
A Lilium Tigrinum Case, . . . . . 241
Poisoning by Tea, . . . . . . . . 355
Saccharum, . . . . . . . . . . . . 334
Proving of Tussilage Petasites, . . 349
Birdsall, A. H., M. D. Verifications, . 189
Bismuth sub. nitrate, . . . . . . . 208, 209
Blind from a Snake Bite. Note, . . . 455

PAGE
Bœnninghausen, Letter of Dr. Nunez to Dr., . . . . . . . . . . . . . . . 323
Bœnninghausen: Relative Worth of Symptoms, with some Remarks on Borax, . . . . . . . . . . . . . . . 392
Bœnninghausen's Treatment of Croup. A. McNeil, M.D., . . . . . . . . . . 138
Boils in the Axillæ, . . . . . . . . . . 4
Books at the Bedside, Use of. E. J. Lee, M. D., . . . . . . . . . . . . . 49
Book Notices and Reviews, . . 48, 111, 167, 215, 263, 309, 357, 401, 454
Borax, . . . . . . . . 121, 208, 336, 339, 392
Borax, some Remarks upon. Bœnninghausen, . . . . . . . . . . . . . . 392
Boston Organon Society, Proceedings of, . . . . . . . . . . . . . 122, 229, 299
Bovista, . . . . . . . . . . . . . . . 300
Breasts, the Care of. Read by Dr. J. B. G. Custis, . . . . . . . . . . . . 386
Breast, a Broken. Frank Kraft, M. D., 414
Brewer's Yeast as a Remedy. S. L., . 351
Bright's Disease, Lectures on. By Robt. Saundby, M. D. Review of, . 309
Bromine, . . . . . . . 81, 181, 184, 201, 251
Bromide of Potassium, . . . . . . . . 20
Bronchitis, Subacute, . . . . . . . . 97
Brooklyn Subscriber, . . . . . . . . . 168
Brownell, W. G., M. D. Is Homœopathy Sufficient in all Cases? . . . . 52
Bryonia—Some Notes—Some Mistakes. H. E. Potter, M. D., . . . . . . . . 239
Bryonia, 137, 179, 192, 239, 245, 251, 305, 314, 316, 338, 357, 417, 425
Buck, M. J., M. D. Homœopathy of the Present as Compared with that of Hahnemann, . . . . . . . . . . 411
Burnett, J. Compton, M. D. On Gonorrhœa in its Constitutional Aspects, 217
Butler, Clarence Willard, M. D. Transverse Presentation: A Case with Remarks, . . . . . . . . . . . . . . . 275

Cactaceæ. E. M. Hale, M. D., . . . . 450
Cactus grand, . . . . . . . . . . . . 208
Calcarea-carb., 19, 82, 144, 149, 150, 208, 210, 247, 316, 323, 339, 340, 419, 425
Calcarea fluorica, . . . . . . . . . . . 189
Cal.-iod, . . . . . . . . . . . . . . 82
Calc phos., . . . . . . . . . . . . . . 82, 209
Calendula, In Praise of. Alice B. Campbell, M. D., . . . . . . . . . . . 298
Campbell, Alice B. In Praise of Calendula, . . . . . . . . . . . . . . . 298
Camphor, . . . . . . . . . . . . . . . 425
Cannabis-indica, . . . . . . . . . . . 426
Cannabis sativa, . . . . . . . . . . . 30, 150
Cantharis, Analytic Study of. Edward Fornias, M. D., . . . . . . . . 152
Cantharis, . . . . . . . 30, 82, 184, 324, 426
Cancer, Dr. Mohr's Case of. C. Mohr, M. D., . . . . . . . . . . . . . 95
Capsic-an., . . . . . . . . . . . . . . 184
Carbolic acid, . . . . . . . . . . . . . 197
Carbo an., . . . . . . . . . . . . . . . 399
Carbo vegetabilis, A Note upon. E. J. Lee, M. D., . . . . . . . . . . . . 100
Carbo Vegetabilis, Therapeutic Observations upon. C. Hering, M. D., 244
Carb-veg., . . . . . . . . . . . . . . . 208
Case for Counsel. Geo. H. Clark, . . 205
Case of Emperor Frederick III. By Edgar S. Werner. Review of, . . . 112
Cases from Practice. Charles L. Swift, M. D., . . . . . . . . . . . . . . . 437

PAGE
Cases, On Reporting. S. L., . . . . . 417
Caulophyllum, . . . . . . . . . . . . 426
Causticum, . . 121, 174, 245, 247, 336, 399, 426
C. C. H. In Memoriam—Edward Bayard, M. D., . . . . . . . . . . . . . . 437
Chamomilla, . . 143, 190, 236, 250, 337, 426
Chelidonium, . . . . . . . . . . . . . 175
China, . . . . . . . . . . . . 101, 426, 436
Chinium sulph., . . . . . . . . . . . . 122
Chronic Cases, Some Practical Hints upon the Management of, . . . . . 313
Chronic Diseases, Intercurrent Remedies for. F. H. Lutze, . . . . . 203
Circuta-vir., . . . . . . . . . . . . . 426
Cimicifuga, . . . . . . . . . . . . . 240, 427
Cina, . . . . . . . . . . . . . . . . 209, 427
Cinnabar Case, A. E. W. Berridge, M. D., . . . . . . . . . . . . . . . 399
Cinnabar, . . . . . . . . . . . . . . . 208
Cistus can., . . . . . . . . . . . . . . 238
Clark, Geo. H., M. D. Case for counsel, 205
Clark, Geo., H., M. D. Notes from past meetings of the Lippe Society, 234
Clark, Geo. H., M. D. Proceedings of the Lippe Society, . . 32, 75, 135, 177, 355
Clark, Geo. H., M. D. Sodium Ethylate, . . . . . . . . . . . . . . . . . 306
Clematis, . . . . . . . . . . . . . . . 209
Clinical Cases. E. W. Berridge, M. D., 146, 192
Clinical Cases. George Logan, M. D., 420
Clinical Cases. Clarence M. Payne, M. D., . . . . . . . . . . . . . 199, 250, 398
Clinical Cases, Some. R. M. Theobold, M. D., . . . . . . . . . . . . . . . 248
Clinical Conversation, A. E. W. Berridge, M. D., . . . . . . . . . . . . 347
Clinical Medicine. G. W. Sherbino, M. D., . . . . . . . . . . . . . . . 419
Clinical Record, The, Notice of, . . . . 168
Clinical Records, The Uses and Abuses of. Edward Cranch, M. D., . 282
Clausen, Daniel W., M. D. Materia Medica, . . . . . . . . . . . . . . . 160
Clausen, Daniel W., M. D. Theridion Curassavicum, . . . . . . . . . . . 162
Close, Stuart, M. D. Totality of Symptoms and Concomitance, . . . . . . 22
Cocculus, . . . . . . . . . . . . . . 149, 247
Coffea, . . . . . . . . . . . . . 203, 366, 427
Cohen, S. W., M. D., Practice, . . . . 342
Colchicum in Gout, . . . . . . . . . 326
Colchicum, . . . . . . . . 82, 161, 197, 316
Collinsonia can., . . . . . . . . . . . 175
Colocynth, . . . . . . . . . . . . . 131, 178
Complimenting Dr. Kent, . . . . 47
Conium, . . . . . . . . . 190, 247, 324, 348
Constipation of Berberis Vulgaris. John L. Ferson, M. D., . . . . . . . 174
Consumptives, Diarrhœa of. F. L. Griffith, M. D., . . . . . . . . . . . 202
Copaiva Balsam, . . . . . . . . . . . 223
Cough Symptom, A. . . . . . . . . . 15
Cranch, Edward, M. D. The Uses and Abuses of Clinical Records, . . . . 282
Cremation of the Dead. Dr. Wm. B. Clarke. Review of, . . . . . . . . 454
Crocus Sativus, . . . . . . . . . . . . 324
Crotalus, . . . . . . . . . . . 82, 326, 427
Croton, . . . . . . . . . . . . . . . . 348
Crot-tig., . . . . . . . . . . . . . . . 26
Croup. L. P. Foster, M. D., . . . . . 201
Croup, Bœnninghausen's Treatment of. P. P. Wells, M. D., . . . . . . . . . 5

PAGE
Croup Powders, Bœnninghausen's. A. McNeil, M. D., . . . . . . . . . 302
Croup, Bœnninghausen and McNeil. P. P. Wells. . . . . . . . . . . . . 214
"Croup," More. W. S. Gee, M. D., . . 250
Cubebs, . . . . . . . . . . . . . . . . . 253
Culex Musca, . . . . . . . . . . . . . 61
Cuprum, . . . . . . . 82, 246, 247, 336, 427
Cuprum-acet., . . . . . . . . . . . . 428
Custis, J. B. G., M. D. The Care of the Breasts, . . . . . . . . . . . . . . . 387
Cyclamen, . . . . . . . . . . . . . . 82
Cyprepedium, . . . . . . . . . . . . 428

Diagnostic Hint, A. . . . . . . . . . 37
Died. Dr. G. Felix Matthes, . . . . . . 215
Digitalis, . . . . . . . . . . . . . . . 66
Dioscorea, . . . . . . . . . . . . . . 83
Dioscorea, Therapeutics of the Throat. James B. Bell, M. D., . . . . . . . . 40
Diphtheria. Horace Still, M. D., . . 182
Diphtheria, treated by Lac-caninum. Sequel to a Case, . . . . . . . . . . 70
Dr. E. M. Hale's Cactaceæ, . . . . . 450
Drosera, . . . . . . . . . . . . . . . 338
Drugs, The Dual Action of. E. J. Lee, M. D., . . . . . . . . . . . . . . . . 3
Dudley Pemberton, M. D. The Institute Session of 1889, . . . . . . . . 109
Dulcamara, . . . . . . . . . . . . . 83, 187

Eaton, Samuel L., M. D. Meddlesome Midwifery, . . . . . . . . . . . . . 139
Editorial Notes. E. J. Lee, M. D., 1, 49, 409
Elaps coral, . . . . . . . . . . 83, 208, 357
Electricity and the Methods of its Employment in Removing Superfluous Hair. By Plym J. Hayes, M. D. Review of, . . . . . . . . . . . . . 263
Electricity in the Diseases of Women, G. Benton Massey, M. D. Review of, 358
Electrical Distribution of Heat, Light, and Power. By Harold P. Brown. Review of, . . . . . . . . . . . . . 405
Electro-Therapeutics, or Electricity in its Relations to Medicine and Surgery. By William Harvey King, M. D. Review of, . . . . . . . . . 263
Errata, . . . . . . . 111, 264, 311, 406, 455
Eupatorium perfol., . . . . . . . 330, 331
Euphrasia, . . . . . . . . . . . . . . 209
Explanation Wanted. P. P. Wells, M. D., . . . . . . . . . . . . . . . . . . 103
Explanation, An. Julius G. Schmitt, 166

Fagopyrum. . . . . . . . . . . . . . 196
Favorite Prescriptions of Distinguished Practitioners, with Notes on Treatment. B. W. Palmer, M. D. Review of, 48
Ferrum aceticum, . . . . . . . . . . 173
Ferrum phos., . . . . . . . . . . 20, 38, 83
Ferson, John L., M. D. Constipation of Berberis Vulgaris, . . . . . . . . 174
Filters and other Means Employed to Purify Drinking Water. Chas. G. Currier, M. D. Review of, . . . . . 310
Fincke, B., M. D. Allopathic Ignorance and Arrogance, . . . . . . . 212
Fistula in Ano. C. C. Howard, M. D., 280
Fluoric acid, . . . . . . . . . . . . . 83
For Sale, . . . . . . . . . . . 264, 311, 456
Fornias, Edward, M. D. Cantharis, . 152
Foster, L. P., M. D. Croup . . . . . 201
Fracture of Rib with aggravation and speedy relief from Hypericum. B. L. B. Baylies, M. D., . . . . . . . 198
Fragmentary Provings. E. W. Berridge, M. D., . . . . . . . . . . . . 60
French, Hayes C., M. D. Homœopathic Prescribing, . . . . . . . . . 21
French Liquers, . . . . . . . . . . . 168

Gee, W. S., M. D. More Croup, . . . 250
Gee, Prof. Questions Asked by Students During a Course of Lectures on the *Organon*, . . . . . . . . . . 169
Gelsemium, . . . . . . 69, 84, 145, 375, 428
Gelsemium. A. McNeil, M. D., . . . . 334
Germania, The ; Notice of, . . . . . . 357
Gleanings from Discussions upon Papers Read at the Recent Meeting of the International Hahnemannian Association, . . . . . . . . . . . . 361
Gemiasma Verdans, . . . . . . . . . 61
Glonoine, . . . . . . . . . . . . . . . 428
Gonorrhœa in its Constitutional Aspects on. Dr. J. Compton Burnett, 217
Gonorrhœa in its Constitutional Aspects on, W. M. James, M. D., . 228
Good Opening, A. Note, . . . . . . . 216
Gout, Colchicum in. B. Simmons, M. D., . . . . . . . . . . . . . . . 326
Graphites, . . . . . . . . . . . 76, 84, 385
Griffith, F. L., M. D. Diarrhœa of Consumptives, . . . . . . . . . . . . . 202
Griffith, F. L., M. D. Nux Vomica, . 210
Guarea-tri, . . . . . . . . . . . . . . 84
Guernsey, Wm. Jefferson, M. D. Mastitis, . . . . . . . . . . . . . . 285
Guernsey's Bœnnninghausen, Review of, . . . . . . . . . . . . . . . . . 406
Guernsey's Diphtheria Card, . . . . . 455
Gymnocladus, . . . . . . . . . . . . 84

Hahnemann's Essay, . . . . . . . . . 159
Hahnemann Club, A New, . . . . . . 455
Hahnemannian Hospital, The Rochester, . . . . . . . . . . . . . 391, 447
Hale, E. M., M. D. Cactaceæ, . . . . 450
Hamamelis. . . . . . . . . . . . . . . 84
Haralson, H. H., M. D. Therapeutic Progress, . . . . . . . . . . . . . . . 443
Hawley, Mrs. Wm. A. Obituary, . . 305
Headache and its Materia Medica, By B. F. Underwood, M. D. Review of, . . . . . . . . . . . . . . . . . 111
Headache and Neuralgia. J. Leonard Corning. Review of, . . . . . . . 112
Heart, the Fatty. Professor E. H. Kisch, . . . . . . . . . . . . . . . . 16
Heath, Alfred. Polypus of Rectum, . 200
Hellebore, . . . . . . . . . . . 247, 248, 428
Helt, L. L., M. D. Homœopathy Triumphant, . . . . . . . . . . . . 449
Helt, L. L., M. D., Married, . . . . . 216
Hepar, Sulphur, 10, 73, 84, 92. 151, 180, 201, 203, 245, 428
Hering's Guiding Symptoms of the Materia Medica, Review of, . . . . 112
Hering, C. Some Practical Remarks, 151
Hering, C., M. D. Therapeutic Observations upon Carbo Vegetabilis, . . . . 244
Holbrook, E. H., M. D. A Case Treated with the Tissue Remedies, . . . . . 38
Holbrook, E. H., M. D. A Kali-phosphoricum Case, . . . . . . . . . . 109
Holmes, H. P., M. D. A Reply to the Criticism of Dr. Holmes, . . . . . . 92

PAGE

Holmes, H. P., M. D. Therapeutics of Convulsions, . . . . . . . . . . . . . 423
Homœopathic International Congress (Note), . . . . . . . . . . . . . . . . 111
Homœopathic Medical Society of State of Penna., Meeting of, . . . . . . . 407
Homœopathic Medicines. The Principal Uses of the Sixteen Most Important and Fourteen Supplementary. E. Gould & Son. Review of, . . . 358
Homœopathic Prescribing. Julius G. Schmitt, M. D., . . . . . . . . . . 120
Homœopathic Remedy, The Repetition of the. Translated from Hahnemann. F. H. Lutze, M. D., . . . . 113
Homœopathy Triumphant. L. L. Helt, M. D., . . . . . . . . . . . . . 449
Homœopathy Again Vindicated. (Note), . . . . . . . . . . . . . . . . 111
Homœopathy of the Present as compared with that of Hahnemann. M. J. Buck, M. D., . . . . . . . . . . . 411
Homœopathy Sufficient in all Cases? Is. W. G. Brownell, M. D., . . . . 52
Hospital, A Homœopathic in Italy, . 257
Hospital, Homœopthic, at Melbourne, Report of, . . . . . . . . . . . . . 48
Hospital, Hahnemannian, at Rochester, . . . . . . . . . . . 48, 391, 447
Howard, C. C., M. D. Fistula in Ano, 280
Hydrargyrum Metallicum, . . . . . . 118
Hydrastis Canadensis. A. McNeil, M. D., . . . . . . . . . . . . . . . 98
Hydrastis, . . . . . . . . . . . . . . . 69
Hydrophobinum, . . . . . . . . . . . . 428
Hydrocyanic Acid, . . . . . . . . . . 428
Hyoscyamus, . . . . . . . . 130, 145, 246, 429
Hypericum. Fracture of a Rib, with Immediate Aggravation and Speedy Relief from. B. L. B. Baylies, . . . 198

Ignatia, . . 67, 85, 181, 184, 239, 247, 249, 337, 429
Indiana Institute of Homœopathy, . . 407
Indigo, . . . . . . . . . . . . . . 248, 429
Indium. Therapeutics of the Throat. James B. Bell, M. D., . . . . . . . . 40
Indium, . . . . . . . . . . . . . . . . 85
International Medical Annual for 1889, Review of, . . . . . . . . . . . . . 215
International Hahnemannian Association, Notice of, . . . 48, 110, 216, 407, 408
I. H. A. Bureau of Materia Medica and Provings. E. A. Ballard, M. D., . . 110
International Hahnemannian Association, Proceedings of the. S. A. Kimball, M. D., . . . . . . . . . . 265
International Hahnemannian Association, The New Board of Censors of the, . . . . . . . . . . . . . . . . 407
Influenza, Epidemic, . . . . . . . . . 456
In Memoriam. Edward Bayard, M. D., . . . . . . . . . . . . . . . . 437
In Memoriam. Dr. Wm. R. Childs, . 41
In Memoriam. Geo. F. Foote, M. D., . 255
In Memoriam. Henry Noah Martin, M. D., . . . . . . . . . . . . . . . . 443
In Memoriam. G. Felix Matthes, . . 256
In Memoriam. David Wilson, M. D., . 441
Iodine, . . . . . . . . . . . . 85, 201, 249
Ipecacuanha. An Unnoticed Symptom Clinically Verified, by Dr. Mossa. S. L., . . . . . . . . . . . . . . . . 211
Ipecac, . . . . . . . . . . . . . . . . 429
Iris versicolor. A. McNeil, M. D., . . 173
Iris vers., . . . . . . . . . . . . . 76, 85, 209
Is Homœopathy Sufficient in All Cases? W. G. Brownell, M. D., . . . . . . . 52

Jacea, . . . . . . . . . . . . . . . . 85
James, W. M., M. D., on Gonorrhœa in its Constitutional Aspects, . . . . 228
Johnstone, R. B. A New Remedy, and a New Indication for an Old One, . . . . . . . . . . . . . . . . 257
Journal of Homœopathics, The, Notice of, . . . . . . . . . . . . . . . . . 216

Kali bichromicum, . . . 27, 85, 91, 173, 185, 201, 251, 331, 336, 355, 421
Kali-brom., . . . . . . . . . . . . 86, 430
Kali-carb., . . . . . . . . . . . . 340, 430
Kali-hyd., . . . . . . . . . . . . . . 75
Kali-muriat., . . . . . . . . . . . . . 86
Kali permang, . . . . . . . . . . . . 185
Kali phosphoricum Case, A. E. H. Holbrook, M. D., . . . . . . . . . . . . 109
Kali-phos., . . . . . . . . . . . . 38, 185
Kalmia, . . . . . . . . . . . . . . . 320
Kimball, S. A., M. D. Proceedings of the Boston *Organon* Society, . . . 63, 68, 122, 187, 299
Kimball, S. A., M. D. Proceedings of the International Hahnemannian Association, . . . . . . . . . . . . 265
Kraft, Frank, M. D. A Broken Breast, 414
Kreosote, . . . . . . . . . . . . . 259, 430

Lac Caninum. S. Swan, M. D., . . . 354
Lac Caninum, . . 29, 86, 185, 249, 273, 285, 416
Lac-can. Sequel to a Case of Diphtheria Treated by, . . . . . . . . . 74
Lac. felinum, . . . . . . . . . . . . . 192
Lachesis Symptom, Verification of. J. E. Russell, . . . . . . . . . . . . 254
Lachesis, . 73, 76, 79, 87, 91, 137, 185, 196, 208, 210, 245, 249, 321, 336, 377, 399, 420, 430, 435
Lachnanthes, . . . . . . . . . . . 186, 326
Lactic Acid, . . . . . . . . . . . . . 147
Lactuca virosa, . . . . . . . . . . 136, 146
Lac Vaccinum. S. Swan, M. D., . . 252
Laurocerasus, . . . . . . . . . . . 209, 430
Lectures upon the Diseases of the Heart. By E. M. Hale, M. D. Review of . . 263
Lectures of Professor Morgan. John Morgan, M. D., . . . . . . . . . . 202
Ledum, . . . . . . . . . . . . . . 417, 419
Lee, E. J., M. D. Acute and Chronic Tonsillitis, . . . . . . . . . . . . . 73
Lee, E. J., M. D. A Case of Typhoid Fever, with Comments, . . . . . . 42
Lee, E. J., M. D. The Dual Action of Drugs, . . . . . . . . . . . . . . . 3
Lee, E. J., M. D. Editorial Notes, . 1, 49
Lee, E. J., M. D. A Note upon Carbo Vegetabilis, . . . . . . . . . . . . 100
Lee, E. J., M. D. A One-sided View, 1
Lee, E. J., M. D. The Repertory, . . 410
Lee, E. J., M. D. Telling the whole Truth, . . . . . . . . . . . . . . . 51
Lee, E. J., M. D. The Use of Books at the Bedside, . . . . . . . . . . . 49
Ledum, . . . . . . . . . . . . . . 319, 324
Legouvé, Ernest. Resurrection of a Child. An Incident of Hahnemann's Practice, . . . . . . . . . . 60
Lilienthal, J. E., M. D. Sanicula, . . 338
Lilienthal, S., M. D. What shall we

PAGE
do to enter the Kingdom of Materia Medica? . . . . . . . . . . . . . . 103
See also " S. L."
Lilium Tigrinum Case, A. E. W. Berridge, M. D., . . . . . . . . . 241
Lilium Tigrinum and Prolapsus Uteri. Thomas G. Roberts, M. D., . . . . . 242
Lippe Society. Notes from past meetings of the. Geo. H. Clark, M. D., . 234
Lippe Society, Proceedings of. Geo. H. Clark, M. D., . . 32, 75, 135, 177, 355
Logan, George, M. D. Clinical Cases, 420
Lutze, F. H., M. D. Antipsoric Remedies. Extract from Bœninghausen's Preface, . . . . . . . . . . . . 119
Lutze, F. H., M. D. Intercurrent Remedies for Chronic Diseases, . . 203
Lutze, F. H., M. D. Remedies for Disturbances of the Antipsoric Cure, . 203
Lutze, F. H., M. D. The Repetition of the Homœopathic Remedy. From German of Hahnemann, . . . . . . 113
Lycopodium, . 4, 87, 121, 144, 150, 178, 180, 186, 237, 245, 247, 251, 325, 326, 347, 374, 430
Lycopus-virg., . . . . . . . . . . . . 15

Magnesia-carb., . . . . . . . . . . 179, 340
Magnesia Muriatica, . . . . . . 20, 149, 175
Magnesia-Phosphoricum, A Proving of the CM Potency by Olfaction, . . 374
Magnesia-phos., . . . . . . . 209, 250, 374
Mancinella, . . . . . . . . . . . . 87
Manual of Dietetics, A. By W. B. Pritchard, M. D., Review of, . . . . . . 167
Married. Dr. L. L. Helt, . . . . . . 216
Mastitis. Wm. Jefferson Guernsey, M. D., . . . . . . . . . . . . . . 285
Mastitis, Its Treatment, with a Repertory, . . . . . . . . . . . . . . . . 385
Materia Medica. Daniel W. Clausen, M. D., . . . . . . . . . . . . . . 160
Materia Medica? What shall we do to enter the Kingdom of. S. Lilienthal, M. D., . . . . . . . . . . 103
Materia Medica. On the Application of the Homœopathic. S. L., . . . 104
Materia Medica and Homœopathic Therapeutics. A Hand Book of. By Timothy F. Allen, M. D. Review of, 358
Matthes, Dr. G. Felix, Death of, . . . 215
McNeil, A., M. D. Bœnninghausen's Treatment of Croup, . . . . . . . . 138
McNeil, A., M. D., Bœnninghausen's Croup Powders, . . . . . . . . . . 302
McNeil, A., M. D. Gelsemium, . . . 334
McNeil, A., M. D. Hydrastis Canadensis, . . . . . . . . . . . . . . . . 98
McNeil, A., M. D. Iris Versicolor, . . 173
McNeil, A. Repetition of the Remedy, 171
McNeil, A., M. D. Spinal Paralysis, . 96
Medical Aid in Parturition. B. L. B. Baylies, M. D., . . . . . . . . . . . 142
Medical Annual, The, Notice of, . . . 216
Melbourne, Report of the Homœopathic Hospital at, Review of, . . . 48
Melilotus alba, . . . . . . . . . . . . 381
Meningitis Cerebro-spinalis Subacuta. S. L., . . . . . . . . . . . . . . . 333
"Mental Derangements." G. W. Sherbino, M. D., . . . . . . . . . . 246
Mercurius, . . . 88, 92, 173, 179, 189, 200, 222, 228, 231, 245, 280, 321, 357, 417
Mercurius cyanatus, . . . . . . . . 88, 186
Mercurius-iod-flavus, . . . . . 88, 186, 210
Mercurius-iod-ruber, . . . . . . . . 88, 186
Mesmerismus, . . . . . . . . . . . 203
Metal Track on Railways. Preliminary Report. E. E. Russell Tratman. Review of, . . . . . . . . . . . 359
Mezereum, . . . . . . . . . . 125, 175, 418
Midwifery, Meddlesome. Samuel L. Eaton, M. D., . . . . . . . . . . . . 139
Millefolium, . . . . . . . . . . . . . 198
Minnesota State Homœopathic Society, 47
Modern Superstition in Disease, The Germ Theory Reconsidered. Lewis Sanders. Review of, . . . . . . . . 404
Mohr, C., M. D. Dr. Mohr's Case of Cancer, . . . . . . . . . . . . . . 95
Morgan, John C., M. D. Prof. Morgan's Lectures, . . . . . . . . . . . 202
Morphia *vs.* Homœopathy. (Note), . . 168
Morrow, H. C. Some Practical Notes, 254
Moschus, . . . . . . . . . . . . . . 430
Murex Purpurea, . . . . . . . . . . . 60
Muriatic Acid, . . . . . . . . . . . . 88

Naja tri, . . . . . . . . . . . . . . 89, 186
Nash, E. B., M. D. A New Potentizer, 106
Natrum mur., . . . 191, 194, 208, 210, 247, 324, 338
Natrum sulphuricum, . . . . . 39, 208, 209
Neuralgia, Intermittent. S. L., . . . 354
Nitric Acid in Injuries to the Spine. B. Simmons, M. D., . . . . . . . . . 327
Nitric Acid, . . . . . . . . . . 89, 186, 223
Notes and Notices, . . . 48, 111, 168, 215, 264, 311, 406, 455
Note, Editors, . . . . . . . . . . . . 15
Note. S. L. . . . . . . . . . . . . . . 353
Notes, Some Practical. H. C. Morrow, 254
Nux Vomica. F. L. Griffith, M. D., . 210
Nux Vomica, . . 30, 143, 178, 191, 203, 208, 210, 247, 248, 324, 430, 435

Obituary. Mrs. William A. Hawley, 305
October and November Numbers, The. (Note), . . . . . . . . . . . . . . . 408
Œnanthe-crocata, . . . . . . . . . 430
Official Health Bulletin, No. 7, of Penn. State Board of Health, Notice of, . . 405
One of Many, . . . . . . . . . . . . . 456
Oneida County Hom. Med. Society, Proceedings of, . . . . . . . . . . . 232
On the Relative Worth of Symptoms, with Some Remarks on Borax, Bœnninghausen, . . . . . . . . . . 392
Ophthalmic Hospital of New York, The Eleventh Annual Announcement of the College of, Notice of, . 311
Opium, . . . 176, 203, 237, 246, 336, 347, 431, 434
*Organon* Society of Boston, Proceedings of. S. A. Kimball, M. D., 63, 68, 187
*Organon*, Questions Asked by Students During a Course of Lectures on, The. Prof. Gee, . . . . . . . . 169
Otorrhœa, . . . . . . . . . . . . . . 97
Ovaritis of the Left Side, Thuja in. B. Simmons, M. D., . . . . . . . . . . 328
Oxalic Acid, A Case of Poisoning with. S. L., . . . . . . . . . . . . 400

Pamphlets Received, Notice of, . . . 167
Parternership. (Note), . . . . . . . . 215
Parturition, Medicinal Aid in. B. L. B. Baylies, M. D., . . . . . . . . . 142
Pastinaca Sat., . . . . . . . . . . . . 207

PAGE
Payne, C. N., M. D. Clinical Cases, . 199, 250, 398
Peculiar Case, A. H. E. Potter, M. D., 240
Petroleum, . . . . . . . . . . . . . . 247
Phosphorus, . . . 8, 19, 34, 89, 150, 197, 202, 246, 248, 377, 431, 437
Phos. ac., . . . . . . . . . . . . . . . 398
Photographic Illustrations of Skin Diseases. Geo. Fox, M. D. Review of, 454
Physicians' Pocket Day-book, Journal, and Ledger, Review of, . . . . . . 455
Phytolacca, . . 89, 92, 186, 209, 273, 285, 416
Plantago, . . . . . . . . . . . . . . . 28
Plumbum, . . . . . . . . . . . . . 90, 176, 431
Podophyl., . . . . . . . . . . . . . 26, 300
Poisoning by Tea. E. W. Berridge, M.D., 355
Polypus of Rectum. Alfred Heath, . 200
Potentizer, A New. E B. Nash, M. D., 106
Potter, H. E., M. D. Bryonia—Some Notes—Some Mistakes, . . . . . . . 239
Potter, H. E., M. D. Clinical Case, Rus Tox, . . . . . . . . . . . . . . 354
Potter, H. E., M. D. Peculiar Case, A. 240
Practical Treatise on Nervous Exhaustion, A. By G. M. Beard, M. D. Review of, . . . . . . . . . . . . . 167
Practice. S. W. Cohen, M. D., . . . . 342
Prescribing. Homœopathic. Hayes C. French, M. D., . . . . . . . . . . . 21
Proceedings of the 24th Annual Session of Ohio Medical Society, Review of, . . . . . . . . . . . . . 167
Provings, Fragmentary. R. M. Theobold. M. D., . . . . . . . . . . . . 253
Psorinum, . . . . . . . . . . . . 44, 90, 180
Psychic Life of Micro-Organisms. Alfred Benet. Review of, . . . . 359
Pscyhology as a Natural Science Applied to the Solution of Occult Psychic Phenomena. By C. G. Raue, M. D. Review of, . . . . . . . . 401
Pulmonaria Sticta. C. Carleton Smith, M. D., . . . . . . . . . . . . . . . 35
Pulsatilla, . . 26, 125, 143, 179, 182, 203, 228, 230, 231, 247, 248, 249, 276, 302, 319, 344, 383, 431

Questions asked by Students during a course of Lectures on the *Organon*. Prof. Gee. . . . . . . . . . . . . . . 169

Ranunculus bulb., . . . . . . . . . . 151
Ranunculus sceleratus, . . . . . . 90, 246
Relation of Homœopathy to Natural Science, Review of. . . . . . . . 454
Remedies for Disturbances of the Antipsoric Cure. F. H. Lutze, . . . . . 203
Remedies? What are the. . . . . . . 258
Remedy, a New, and a New Indication for an Old One. R. B. Johnstone, M. D., . . . . . . . . . . . . 257
Removals, . 48, 111, 168, 215, 264, 406, 456
Repertory, The. E. J. Lee, M. D., . . 410
Repertory to Hering's Condensed Materia Medica, Review of, . . . . . 405
Repertory to Labor and After Pains. John V. Allen, M. D., . . . . . . . 291
Repetition of the Remedy. A. McNeil, 171
Reply to the Criticisms of Dr. Holmes. H. P. Holmes, M. D., . . . . . . . . 92
Report on the Forest Conditions of the Rocky Mountains. B. E. Fernow. Review of, . . . . . . . . . . . . 360
Reporting Cases, On. S. L. . . . . . 417
Resolutions of Homœopathic Union, 440

PAGE
Resurrection of a Child. An incident of Hahnemann's Practice. Ernest Legouvé, . . . . . . . . . . . . . 60
Rhus-tox, . . 90, 96, 146, 179, 180, 194, 236, 247, 249, 250, 251, 269, 305, 319, 330, 336, 344, 364, 437
Rhus-tox, Clinical Case. H. E. Potter, M. D., . . . . . . . . . . . . . . . 354
Roberts, Thomas G., M. D. Lilium Tigrinum and Prolapsus Uteri, . . 242
Rochester Hahnemannian Hospital, The, . . . . . . . . . . . . . 48, 391, 447
Rochester Hahnemannian Society, Proceedings of the, . . . . . 25, 72, 132
Russell, J. E., M. D. Verification of a Lachesis Symptom, . . . . . . . . . 254
Ruta-grav., . . . . . . . . . . . . . . 208

Sabadilla, . . . . . . . . . . . . . . 90
Sabina, . . . . . . . . . . . . 140, 210, 389
Saccharum. E. W. Berridge, M. D., 334
Sambucus, . . . . . . . . . . . . . . 201
Sanguinaria, . . . . . . . . 90, 108, 208, 209
Sanicula. J. E. Lilienthal, M. D., . 338
Sanicula, . . . . . . . . . . . . 197, 255, 379
Sarsaparilla, . . . . . . . . . . . . . 147
Sciatica. B. Simmons, M. D., . . . . 258
Schmitt, Julius G., An Explanation, 166
Schmitt, Julius G., Homœopathic Prescribing, . . . . . . . . . . . . . . 120
Schmitt, J. G., M. D. Report of the Rochester Hahnemannian Society, 132
*Scientific American, The*, . . . . . . . 168
Scilla, . . . . . . . . . . . . . . . . 236
Secale Cornutum, . . . . . . . . 145, 431
Semen Tiglii. An involuntary proving of. S. L., . . . . . . . . . . . 352
Sepia, . . 149, 173, 177, 192, 195, 229, 247, 323, 383, 390
Sherbino, G. W., M. D. Baptisia Tinctoria, . . . . . . . . . . . . . . . . 330
Sherbino, G. W., M. D. Clinical Medicine, . . . . . . . . . . . . . . . . 419
Sherbino, G. W., M. D. "Mental Derangements," . . . . . . . . . . . . 246
Silicea, . . . . . . . . . 90, 324, 336, 420, 431
Simmons, B., M. D. Colchicum in Gout, . . . . . . . . . . . . . . . . . 326
Simmons, B., M. D. Nitric Acid in Injuries to the Spine, . . . . . . . . . 327
Simmons, B., M. D. Sciatica, . . . . 258
Simmons, B., M. D. Thuja in Ovaritis of the Left Side, . . . . . . . . . . 328
Simmons, B., M. D. Torticollis, . . . 325
S. L. Brewer's Yeast as a Remedy, . . 351
S. L. A Case of Poisoning with Oxalic Acid, . . . . . . . . . . . . . . . . 400
S. L. Intermittent Neuralgia, . . . . 354
S. L. An Involuntary proving of Semen Tiglii, . . . . . . . . . . . . . 352
S. L. Meningitis Cerebro Spinalis Subacuta, . . . . . . . . . . . . . . . 333
S. L. Note, . . . . . . . . . . . . . . 353
S. L. On Reporting Cases, . . . . . . 417
S. L. On the Application of the Homœopathic Materia Medica, . . . . 104
S. L. An Unnoticed Sympton of Ipecacuanha Clinically Verified by Dr. Mossa, . . . . . . . . . . . . . . . 211
Smith, C. Carleton, M. D. Sticta Pulmonaria, . . . . . . . . . . . . . . 35
Smith, C. Carleton. M. D. Some Points on Tonsillitis, . . . . . . . . . . . 91
Smith, Oran W., M. D. Verifications, . . . . . . . . . . . . . . . . . 207

PAGE
Sodium Ethylate. George H. Clark, M. D., . . . . . . . . . . . . . . 306
Some Practical Remarks. C. Hering, M. D., . . . . . . . . . . . . . . . 151
Southern Journal of Homœopathy, Notice of, . . . . . . . . . . . . . . 216
Spigelia, . . . . . . . . . . . . 148, 190, 208
Spinal Paralysis. A. C. McNeil, M. D. 96
Spine, Nitric Acid in Injuries to the. B. Simmons, M. D., . . . . . . . . . 327
Spongia, . . . . . . . . . . . 8, 10, 180, 201
Stannum, . . . . . . . . . . . . . . 34, 431
Staph., . . . . . . . . . . . . . . . 131, 210
Sticta, . . . . . . . . . . . . . . . . . 33
Sticta Pulmonaria. C. Carleton Smith, M. D., . . . . . . . . . . . . . . . 35
Still, Horace, M. D. Diphtheria, . . . 182
Stramonium, . . . . . 45, 246, 247, 336, 432
Subscriber, A Wide Awake, . . . . . 48
Sulphur and Lycopodium. (Note), . 74
Sulphur, . . . 44, 90, 97, 116, 147, 151, 186, 194, 197, 200, 228, 237, 245, 247, 249, 253, 274, 324, 341, 386, 419, 422, 432
Sulphuric Acid, . . . . . . . . . . 186, 347
Sunstroke, A Case of. E. W. Berridge, M. D., . . . . . . . . . . . . . . . . 451
Surgery. Bureau of, I. H. A. . . . . 39
Surgical Journal, . . . . . . . . . . . 456
Swan, S., M. D. Proving of Lac caninum . . . . . . . . . . . . . . . 354
Swan, S., M. D. Lac vaccinum. . . . 252
Swan, S., M. D. A Proving of Ustilago Maidis, . . . . . . . . . . . . . 253
Swift, Charles L., M. D. Cases from Practice, . . . . . . . . . . . . . . . 435
Symptoms and Concomitance, Totality of. Stuart Close, M. D. . . . . . . . 22

Tabacum, . . . . . . . . . . . . . . 207, 246
Tarentula, . . . . . . . . . . . . . 165, 432
Tartar Emetic, . . . . . . . . . . . . . 432
Telling the Whole Truth. E. J. Lee, M. D. . . . . . . . . . . . . . . . . 51
Terebith, . . . . . . . . . . . . . . 197, 432
Testimonial to J. T. Kent, M. D., . . 108
Therapeutic Methods. By Jabez Dake, M. D. Review of, . . . . . . . . . 167
Therapeutic Progress. H. H. Haralson, M. D., . . . . . . . . . . . . . 443
Therapeutics of Convulsions. H. P. Holmes, M. D., . . . . . . . . . . . . 423
Therapeutics of Nervous Diseases, Review of. Chas. Porter Hart, M. D., 455
Theobald, R. M., M. D. Fragmentary Provings, . . . . . . . . . . . . . . 253
Theobald, R. M., M. D. Some Clinical Cases, . . . . . . . . . . . . . . . 248
Theridion Curassavicum. Daniel W. Clausen, M. D., . . . . . . . . . . . 162
Theridion cur., . . . . . . . . . . . 135, 166
Throat, Therapeutics of the. James B. Bell, M. D., . . . . . . . . . . . . . 40
Thuja in Ovaritis of the Left Side. B. Simmons, M. D., . . . . . . . . . . 328
Thuja, . . . 26, 191, 197, 208, 210, 223, 242, 257, 399, 451

PAGE
Tissue Remedies. A Case Treated with the. E. H. Holbrook, M. D., . . . . 88
To Builders and Those Who Contemplate Building, . . . . . . . . . . 407
Tonsillitis, Some Points on. C. Carleton Smith, M. D., . . . . . . . 91
Torticollis. B. Simmons, M. D., . . . 325
Totality of the Symptoms. Stuart Close, M. D., . . . . . . . . . . . . 22
Transactions of the American Institute. Review of, . . . . . . . . . . 451
Transactions of the International Hahnemannian Association for 1888, Review of, . . . . . . . . . . . . . 112
Transactions of the Mississippi State Med. Asso., . . . . . . . . . . . . . 454
Transverse Presentation. A Case with Remarks. Clarence Willard Butler, M. D., . . . . . . . . . . . . . . . 275
Transverse Presentation. A Case with Some Remarks, . . . . . . . . . . 381
Tuberculinum, . . . . . . . . . . . . 70
Tussilago Petasites, Proving of. E. W. Berridge, M. D., . . . . . . . . . . 349
Typhoid Fever with Comments, A Case of. E. J. Lee, M. D., . . . . . . . . 42

Uses and Abuses of Clinical Records. Edward Cranch, M. D., . . . . . . 282
Ustilago Maidis. A Proving of. S. Swan, M. D., . . . . . . . . . . . . 253

Veratrum. . . . . . . 246, 247, 324, 432, 436
Verat-viride, . . . . . . . . . . . . . 432
Verification, A. E. A. Ballard, M. D., . 259
Verifications. A. H. Birdsall, M. D., 189
Verifications. Oran W. Smith, . . . 207
Verifications of Death, The. (Note), . 264
Vespa, . . . . . . . . . . . . . . . . 207
View, A One-sided. E. J. Lee, M. D., 1
Vipera acuat. car., . . . . . . . . . . 357
Viscum-alb., . . . . . . . . . . . . . 433
Vitrum, . . . . . . . . . . . . . . . . 257

Wells, P. P., M. D. Bœnninghausen's Treatment of Croup, . . . . . . . . 5
Wells, P. P., M. D. Croup, Bœnninghausen and McNeil, . . . . . . . . 214
Wells, P. P., M. D. Explanation Wanted, . . . . . . . . . . . . . . 103
What is Contagion, . . . . . . . . . . 361
What is a Homœpath? . . . . . . . . 453
What are the Remedies? . . . . . . . 408
What Produces Death? . . . . . . . . 452
What Shall we do to Enter the Kingdom of Materia Medica? S. Lilienthal, M. D., . . . . . . . . . . . . 103
Wigg. Dr. George. Extract from Address, . . . . . . . . . . . . . . 259

Zinc, . . . . . . . . . . . . . 91, 98, 247, 433
Zincum Cyanat, . . . . . . . . . . . 333
Zizea, . . . . . . . . . . . . . . . . 433

VOL. IX. DECEMBER, 1889. NO. 12.

### THE HOMŒOPATHIC PHYSICIAN,

A Monthly Journal of Homœopathic Materia Medica and Clinical Medicine.

*"He is a freeman whom truth makes free, and all are slaves beside."*
*"Seek the Truth: come whence it may, cost what it will."*

EDITED AND PUBLISHED BY

EDMUND J. LEE, M. D.,

AND

WALTER M. JAMES, M. D.

NOTICE.

*All articles for publication, books for review, business communications, etc., must be sent to Editors, 1123 Spruce Street, Philadelphia.*

*Subscribers will please notice that this Journal will be sent until arrears are paid and it is ordered discontinued.*

PHILADELPHIA:

No. 1123 SPRUCE STREET.

LONDON
ALFRED HEATH & CO., SOLE AGENTS FOR GREAT BRITAIN,
114 Ebury Street, Eaton Square.

☞ *Yearly Subscription, $2.50, invariably in advance. Single numbers, 25 cts.*

*Single numbers containing the Repertory Supplement, 50 cts.*

Entered at the Philadelphia Post-Office as Second-Class Mail Matter.

### *NOTICE TO SUBSCRIBERS.*

***In order to settle up their accounts, the editors would be greatly obliged to receive prompt payment from all subscribers who are in arrears.***

***The delay in issuing the Repertory number (the combined issue for October and November) and the regular number for December has been unavoidable, and is greatly regretted by the editors.***

---

### CONTENTS.

***MISCELLANEOUS.*** PAGE

Announcement for 1890, . . . . . . . . . . . . 409

The Repertory, . . . . . . . . . . . . . 410

Homœopathy of the Present as compared with that of Hahnemann. Dr. M. J. Buck, . . . . . . . . . . . . 411

A Broken Breast. Frank Kraft, M. D., . . . . . . . 414

On Reporting Cases. S. L., . . . . . . . . . . 417

Clinical Medicine. G. W. Sherbino, M. D., . . . . . . 419

Clinical Cases. George Logan, M. D., . . . . . . . 420

Therapeutics of Convulsions. H. P. Holmes, M. D., . . . . . 423

Cases from Practice. Chas. L. Swift, M. D., . . . . . . 435

In Memoriam—Edward Bayard, M. D., . . . . . . . 437

Resolutions of N. Y. Homœopathic Union, . . . . . . 440

In Memoriam—David Wilson, M. D., . . . . . . . 441

In Memoriam—Henry Noah Martin, M. D., . . . . . . 443

Therapeutic Progress. H. H. Haralson, M. D., . . . . . . 443

The Hahnemann Homœopathic Hospital, of Rochester, N. Y., . . 447

Homœopathy Triumphant. L. L. Helt, M. D., . . . . . . 449

Dr. E. M. Hale's Cactaceæ, . . . . . . . . . . . 450

A Case of Sunstroke. E. W. Berridge, M. D., . . . . . . 451

What Produces Death, . . . . . . . . . . . 452

What is a Homœopath, . . . . . . . . . . . 453

***BOOK NOTICES AND REVIEWS.***

